# Considering multifetal pregnancy reduction in triplet pregnancies: do we forget the emotional impact on fathers? A qualitative study from The Netherlands

**DOI:** 10.1093/humrep/dead275

**Published:** 2024-01-10

**Authors:** P M van Baar, W F J Grijzenhout, M A de Boer, C J M de Groot, E Pajkrt, B F P Broekman, M G van Pampus

**Affiliations:** Department of Obstetrics and Gynecology, Amsterdam UMC, Vrije Universiteit, Amsterdam, The Netherlands; Amsterdam Reproduction and Development Research Institute, Amsterdam UMC, Amsterdam, The Netherlands; Vrije Universiteit, Amsterdam, The Netherlands; Department of Obstetrics and Gynecology, Amsterdam UMC, Vrije Universiteit, Amsterdam, The Netherlands; Amsterdam Reproduction and Development Research Institute, Amsterdam UMC, Amsterdam, The Netherlands; Amsterdam Reproduction and Development Research Institute, Amsterdam UMC, Amsterdam, The Netherlands; Department of Obstetrics and Gynecology, Amsterdam UMC, Universiteit van Amsterdam, Amsterdam, The Netherlands; Amsterdam Reproduction and Development Research Institute, Amsterdam UMC, Amsterdam, The Netherlands; Department of Obstetrics and Gynecology, Amsterdam UMC, Universiteit van Amsterdam, Amsterdam, The Netherlands; Department of Psychiatry, OLVG, Amsterdam, The Netherlands; Amsterdam Public Health Institute, Mental Health Program, Amsterdam UMC, Vrije Universiteit, Amsterdam, The Netherlands; Department of Obstetrics and Gynecology, OLVG, Amsterdam, The Netherlands

**Keywords:** counselling, decision-making, fathers, multifetal pregnancy reduction, multiple pregnancy, triplet pregnancy, psychological impact, psychology

## Abstract

**STUDY QUESTION:**

What factors influence the decision-making process of fathers regarding multifetal pregnancy reduction or maintaining a triplet pregnancy, and how do these decisions impact their psychological well-being?

**SUMMARY ANSWER:**

For fathers, the emotional impact of multifetal pregnancy reduction or caring for triplets is extensive and requires careful consideration.

**WHAT IS KNOWN ALREADY:**

Multifetal pregnancy reduction is a medical procedure with the purpose to reduce the number of fetuses to improve chances of a healthy outcome for both the remaining fetus(es) and the mother, either for medical reasons or social considerations. Aspects of the decision whether to perform multifetal pregnancy reduction have been rarely investigated, and the impact on fathers is unknown.

**STUDY DESIGN, SIZE, DURATION:**

Qualitative study with semi-structured interviews between October 2021 and February 2023.

**PARTICIPANTS/MATERIALS, SETTING, METHODS:**

Fathers either after multifetal pregnancy reduction from triplet to twin or singleton pregnancy or ongoing triplet pregnancies 1–6 years after the decision were included. The interview schedule was designed to explore key aspects related to (i) the decision-making process whether to perform multifetal pregnancy reduction and (ii) the emotional aspects and psychological impact of the decision. Thematic analysis was used to identify patterns and trends in the father’s data. The process involved familiarization with the data, defining and naming themes, and producing a final report. This study was a collaboration between a regional secondary hospital (OLVG) and a tertiary care hospital (Amsterdam University Medical Center, Amsterdam UMC), both situated in Amsterdam, The Netherlands.

**MAIN RESULTS AND THE ROLE OF CHANCE:**

Data saturation was achieved after 12 interviews. Five main themes were identified: (i) initial responses and emotional complexity, (ii) experiencing disparities in counselling quality and post-decision care, (iii) personal influences on the decision journey, (iv) navigating parenthood: choices, challenges, and emotional adaptation, and (v) shared wisdom and lessons. For fathers, the decision whether to maintain or reduce a triplet pregnancy is complex, in which medical, psychological but mainly social factors play an important role. In terms of psychological consequences after the decision, this study found that fathers after multifetal pregnancy reduction often struggled with difficult emotions towards the decision; some expressed feelings of doubt or regret and were still processing these emotions. Several fathers after an ongoing triplet had experienced a period of severe stress in the first years after the pregnancy, with major consequences for their mental health. Help in emotional processing was not offered to any of the fathers after the decision or birth.

**LIMITATION, REASONS FOR CAUTION:**

While our study focuses on the multifetal pregnancy reduction process in the Amsterdam region, we recognize the importance of further investigation into how this process may vary across different regions in The Netherlands and internationally. We acknowledge the potential of selection bias, as fathers with more positive experiences might have been more willing to participate. Caution is needed in interpreting the role of the mother in the recruitment process. Additionally, the time span of 1–6 years between the decision and the interviews may have influenced emotional processing and introduced potential reporting bias.

**WIDER IMPLICATIONS OF THE FINDINGS:**

The emotional impact of multifetal pregnancy reduction or caring for triplets is significant, emphasizing the need for awareness among caregivers regarding the emotional challenges faced by fathers. A guided trajectory might optimize the decision-making and primarily facilitate the provision of appropriate care thereafter to optimize outcomes around decisions with potential traumatic implications.

**STUDY FUNDING/COMPETING INTEREST(S):**

This study received no funding. The authors have no conflicts of interest to declare.

**TRIAL REGISTRATION NUMBER:**

N/A.

## Introduction

Multifetal pregnancies pose unique challenges due to increased risks of perinatal and maternal morbidity and mortality compared to singleton pregnancies (The ESHRE Capri Workshop Group, 2000; American College of Obstetricians and Gynecologists, 2014), particularly in triplet and higher-order pregnancies (Wen *et al.*, 2004; Luke and Brown, 2008; Stone and Kohari, 2015). Complications such as hypertensive disorders of pregnancy and preterm birth are more prevalent in triplet pregnancies compared to twin and singleton pregnancies, highlighting the importance of effective care strategies (Ziadeh, 2000; Day *et al.*, 2005). Parents of multiples face elevated risks of mental health problems related to these pregnancies and associated complications, including emotional distress and compromised quality of life (Garel *et al.*, 1997; Wenze *et al.*, 2015), reflected by higher rates of divorce among parents of multiples (Jena *et al.*, 2011).

When a triplet or higher-order multifetal pregnancy occurs, multifetal pregnancy reduction (MFPR) can be considered. MFPR is defined as a surgical procedure to reduce the total number of fetuses by one or more (Berkowitz and Lynch, 1990; Berkowitz *et al.*, 1996). According to the ACOG guideline of 2017 (American College of Obstetricians and Gynecologists’ Committee on Ethics, 2017), parents facing a triplet or higher-order multifetal pregnancy should receive comprehensive counselling that addresses potential benefits and risks of both MFPR or maintaining the multifetal pregnancy, MFPR procedure details, and emotional consequences of the decision. In the decision-making process whether to perform MFPR, the risk of the procedure (i.e. 7–10% chance of miscarriage (Evans *et al.*, 2001; Chaveeva *et al.*, 2013; van de Mheen *et al.*, 2014; Anthoulakis *et al.*, 2017)) has to be balanced against the possible medical complications associated with a multifetal pregnancy. Additionally, moral, religious, social, cultural, and economic factors play a unique role (American College of Obstetricians and Gynecologists, 2007).

While in quadruplet pregnancies the risks of MFPR are generally considered to outweigh the benefits, in triplet pregnancies there is still no clear consensus whether reduction to a lower-order pregnancy contributes to a decreased risk of medical complications during and after pregnancy (American College of Obstetricians and Gynecologists, 2014; van de Mheen *et al.*, 2014, 2015; Zipori *et al.*, 2017; Evans *et al.*, 2022; Kristensen *et al.*, 2023). Moreover, the psychological aspects of the decision whether to perform MFPR have rarely been investigated, with only a few studies published in the early 2000s (Kanhai *et al.*, 1994; McKinney *et al.*, 1995; Schreiner-Engel *et al.*, 1995; Garel *et al.*, 1997; Bergh *et al.*, 1999). Most of these studies considered the experiences of mothers (McKinney *et al.*, 1995; Schreiner-Engel *et al.*, 1995; Garel *et al.*, 1997) or couples (Kanhai *et al.*, 1994; Bergh *et al.*, 1999), with no exclusive investigation of fathers’ perspectives. It is important for clinicians to understand both mothers’ and fathers’ considerations in the decision whether to perform MFPR and to provide accurate information to both parents about the psychological consequences of the decision. Therefore, we aimed to qualitatively investigate the decision-making process and psychological impact of MFPR or maintaining a triplet pregnancy in fathers.

## Materials and methods

### Setting

Qualitative study with semi-structured interviews between 12 October 2021 and 22 February 2023. This study was a collaboration between a regional secondary hospital (OLVG) and a tertiary care hospital (Amsterdam University Medical Center, Amsterdam UMC), both situated in Amsterdam, The Netherlands.

In The Netherlands, recent centralization effects have focused on improving outcomes of MFPR procedures, ensuring a standardized approach. At Amsterdam UMC, all parents of triplet pregnancies are invited for a counselling appointment prior to the decision whether to undergo MFPR. Parents are informed about the risks associated with both MFPR and maintaining the triplet pregnancy, the possibility of losing the pregnancy, as well as emotional consequences of the decision and potential feelings of guilt and regret that may arise after the procedure. A consultation with a hospital social worker is mandatory to assist in the decision-making, and in The Netherlands, these hospital social workers can also provide support for long-term psychological concerns that might occur. In our study cohort, most counselling conversations and all MFPR procedures are conducted by one trained Gynaecologist, ensuring a standardized approach as well as neutral counselling (i.e. no preference of continuing triplets over MFPR).

### Participants and recruitment

Eligible participants were recruited according to the following approach: in 2021, all women with a triplet pregnancy at Amsterdam UMC within the past 1–6 years, whether they had opted for MFPR or chose to maintain the triplet pregnancy, were invited via postal mail or email to participate in a study focusing on experiences of mothers (i.e. not the present study). All these women were kindly requested to encourage the fathers to take part in the present study. The recruitment process continued in 2022, including additional eligible participants from the preceding year. The selection of a 1- to 6-year time span aimed to incorporate participants who had encountered the decision as recently as feasible, while also ensuring that the decision was made more than a year ago to mitigate the potential influence of recency bias.

Exclusion criteria were age <18 years, no understanding of the Dutch language, objection to use data, living abroad, and if MFPR was performed because of a congenitally abnormal fetus (i.e. selective reduction) or serious maternal medical indication. Purposive sampling (i.e. selection of participants based on predetermined criteria) was not used in this study in accordance with the privacy statement legislation in The Netherlands, ensuring participant privacy and data protection. Recruitment continued until data saturation was achieved, operationalized by the point at which no new information or insights emerged from additional interviews, as determined by the research team.

### Data collection

Data were collected via semi-structured face-to-face interviews. After obtaining informed consent and before the interview, all fathers completed a demographic questionnaire and the Hospital Anxiety and Depression Scale (HADS) online, using Castor EDC (2019), in order to detect any psychological symptoms at the time of the interview that could potentially influence recall bias. The HADS is a screening instrument consisting of 14 questions assessing symptoms related to anxiety and depression disorders (Zigmond and Snaith, 1983). It includes separate subscores for anxiety (HADS-A) and depression (HADS-D), with a subscale score of ≥8 indicating a higher likelihood of experiencing significant symptoms. In case of deviations (i.e. subscale score ≥8), fathers were not precluded from participating. Such deviations were discussed with the participants. If, during the interview, it was determined that psychological symptoms were significantly impacting their daily life and no prior psychological support was in place, the option to contact the general practitioner for arranging psychological support was offered.

### Interviews

All interviews were held by two female authors (P.M.v.B. and M.G.v.P.) with the assistance of a student mastering in Medicine (W.F.J.G.) (Supplementary Table S1) and took place at OLVG (the hospital where the interviewers work) or at the participants’ home address. P.M.v.B. is currently pursuing a PhD in obstetrics and underwent a specialized training in qualitative research and interview techniques. M.G.v.P. is a gynecologist with a broad background in the psychological aspects of obstetrics. None of the interviewers were involved in the prenatal care of the participants. Throughout the study, the interviewers engaged in reflective discussions to minimize the impact of their backgrounds on the research outcomes to uphold the integrity of the findings. At the outset of each interview, all participants were apprised of the purpose and motivations behind the research topic.

As a guide for the semi-structured interviews, several predefined questions were used (Table 1). A pilot test was not conducted for this study. All interviews were audiotape recorded with the participants’ consent. Verbatim transcription was used to transcribe all audio-recorded interviews into anonymized reports with the use of ATLAS.ti. The transcripts were not returned to participants for comments and/or corrections.

**Table 1.** Questions used for semi-structured interviews.

**Decision-making process**
1. How did you make a decision?
2. Are there any specific factors that played an important role in the decision?
3. Were there people involved?
4. How did you experience the pregnancy after the decision?
5. How satisfied were you with the information that was provided, prior to the decision?
6. For the MFPR group: were you aware of the risk of losing the whole pregnancy as a result of the procedure?
6. For the ongoing triplet group: were you aware of the possible risks of maintaining a triplet pregnancy?
7a. How do you look back on the decision?
7b. With today’s knowledge, would you have made the same decision?
**Emotional aspects/psychological consequences**
1. Were you as parents on the same page in the decision-making process?
2. Do you think your opinion mattered?
3. Do you ever regret your decision?
4. Are there any emotional consequences of the decision?
5. Are there any relational consequences of the decision?
6. Are there any other consequences of the decision?
7. How satisfied were you with the guidance/support during the decision-making process and thereafter?
8. During and after the decision, were there any specific people you could go to?
9. What can you recommend to future parents facing the decision?

MFPR, multifetal pregnancy reduction.

All fathers were contacted by telephone within two weeks after their interview, to evaluate their experience of participating in the study. This follow-up also served to assess any psychological discomfort and to determine if additional professional care or follow-up was required. If so, the general practitioner was contacted to facilitate a referral to a healthcare provider.

### Data analysis

Thematic analysis was used to identify trends in the father’s data (Clarke *et al.*, 2015) and was performed in six stages: (i) familiarization with the data, (ii) generating initial codes, (iii) searching for themes, (iv) reviewing potential themes, (v) defining and naming themes and subthemes, and (vi) producing the report. During the coding process, two, and in some cases three, authors (P.M.v.B. and W.F.J.G. and/or M.G.v.P.) independently reviewed and coded all transcripts (Supplementary Table S1). Subsequently, they collaboratively organized the codes into thematic families. To ensure traceability and reliability in theme development, all identified themes and subthemes underwent thorough discussions involving four authors (P.M.v.B., W.F.J.G., B.F.P.B., and M.G.v.P.). Illustrative quotes were used to give adequate descriptions according to ‘thick description’: detailed and specific descriptions of relevant circumstances, situations, and behaviours (Geertz, 1973).

### Ethical approval

The study protocol was reviewed and approved by the Institutional Review Board of the VU University Medical Center (METc VUmc 2020.406). This article complies with the COREQ checklist for reporting qualitative studies (Tong *et al.*, 2007).

## Results

A total of 55 triplet pregnancies were identified during the study period. The response rate was 42%. In these 23 cases, the mothers agreed to invite the father to participate in the present study; 18 after an ongoing triplet pregnancy, 4 after MFPR from triplet to twin pregnancy, and 1 after MFPR from triplet to singleton pregnancy. Data saturation was achieved after 12 interviews, as signified by the absence of any new information being raised. Interview time ranged from 29 to 66 min (mean 48 min).

Some relevant characteristics of the participants are found in Table 2. We included four fathers (33%) in the MFPR group and eight (67%) in the ongoing triplet pregnancy group. This ratio reflects the prevailing distribution of MFPR procedures conducted in triplet pregnancy at Amsterdam UMC. In the MFPR group, three cases involved MFPR from trichorionic triamniotic triplet to twin pregnancies, and one case involved MFPR from a dichorionic triamniotic triplet to a singleton pregnancy. Spontaneous conception accounted for 25% triplet pregnancies, while 75% resulted from intra-uterine insemination or ovulation induction. Three fathers of the ongoing triplet group had an additional (older) child (not reported in Table 2). Three fathers reported a history of psychological problems and received treatment of a psychologist or psychiatrist in the past: one in the MFPR group and two in the ongoing triplet group. The majority of the fathers (92%) scored <8 on both the anxiety and depression subscales of the HADS, indicating no anxiety or depressive symptoms at the time of the interview. The relationship status of all fathers was unchanged compared to the moment of conception. Details on pregnancy outcome are found in Table 3. Nine interviews were conducted at OLVG and three at the fathers’ home address.

**Table 2.** Characteristics of included fathers.

Study information		Pregnancy details		Reported psychological health state					Relationship status
Participant	Study group	Conception	Year of conception	Psychological history	Psychotherapy	Major life events with psychological impact		HADS^1^	
						Unrelated to the pregnancy	Related to the pregnancy		Unchanged^a^
**P1.**	MFPR 3–>2	IUI	2020	Anxiety and depression	+	Troubled childhood, quitted his own business	–	A10D5	Yes
**P2.**	Ongoing triplet	OI	2019	–	−	–	–	A1D2	Yes
**P3.**	Ongoing triplet	IUI	2017	–	−	–	Repeated miscarriages	A1D7	Yes
**P4.**	Ongoing triplet	Spontaneous	2018	Depressive symptoms	+	–	Moment of establishing the triplet pregnancy, birth of triplets	A6D3	Yes
**P5.**	MFPR 3–>2	IUI	2018	–	−	Moving out/renovation, illness and loss of mother in law	–	A6D1	Yes
**P6.**	MFPR 3–>2	IUI	2017	–	−	–	–	A0D0	Yes
**P7.**	Ongoing triplet	OI	2017	–	−	–	–	A1D1	Yes
**P8.**	Ongoing triplet	IUI	2021	ADHD	+	–	Pregnancy complicated by hyperemesis gravidarum resulting in a disabling condition of mother and mental overload of father	A3D4	Yes
**P9.**	Ongoing triplet	Spontaneous	2021	–	−	–	–	A2D2	Yes
**P10.**	Ongoing triplet	OI	2019	–	−	Moving from abroad to The Netherlands at the age of 12	Long period of caring after birth as a result of long hospital stay of mother and neonates at NICU; thereafter experiencing difficulty in changing the caring role into a partner/father role	A1D7	Yes
**P11.**	MFPR 3–>1	Spontaneous	2020	–	−	Divorce of parents, loss of mother and brother in law	–	A2D0	Yes
**P12.**	Ongoing triplet	IUI	2016	–	−	Loss of mother	–	A6D3	Yes

a Permanent relationship/living together or married with mother of child(ren).

1 HADS, A and D show scores for anxiety and depression, respectively, a score of ≥8 indicates a higher likelihood of significant symptoms.

ADHD, attention deficit hyperactivity disorder; HADS, Hospital Anxiety and Depression Scale; IUI, intra-uterine insemination; MFPR, multifetal pregnancy reduction; NICU, neonatal intensive care unit; OI, ovulation induction.

**Table 3.** Pregnancy outcome.

Participant	Study group	GA at birth (weeks^+days^)	Mode of birth	Complications during pregnancy or after birth	Birthweight (grams)			Surviving children
					Child 1	Child 2	Child 3	
**P1.**	MFPR 3–>2	37^+0^	Planned CS	Anemia in pregnancy	2808	2472	–	2/2
**P2.**	Ongoing triplet	33^+5^	Planned CS	GDM, PTB	1709	1988	2200	3/3
**P3.**	Ongoing triplet	35^+6^	Planned CS	GDM, PTB	1895	2010	2455	3/3
**P4.**	Ongoing triplet	34^+0^	Planned CS	PE, FGR, GDM, PTB	1186	1384	1366	3/3
**P5.**	MFPR 3–>2	37^+5^	Planned CS	–	3214	3262	–	2/2
**P6.**	MFPR 3–>2	33^+6^	VD	Severe HG, PTB	2050	2110	–	2/2
**P7.**	Ongoing triplet	33^+1^	Planned CS	PTB	1679	1825	1720	3/3
**P8.**	Ongoing triplet	30^+0^	Planned CS	Severe HG, PTB	1430	1450	1290	3/3
**P9.**	Ongoing triplet	34^+2^	Planned CS	FGR, PTB, PPH	2300	2000	1620	3/3
**P10.**	Ongoing triplet	32^+2^	Emergency CS	PE, FGR, GDM, PTB, postpartum admission maternal intensive care unit due to renal insufficiency (HUS)	1530	1270	1291	3/3
**P11.**	MFPR 3–>1	38^+0^	VD	GH, GDM	3490	–	–	1/1
**P12.**	Ongoing triplet	31^+1^	Planned CS	PTB	1385	1245	1545	3/3

CS, cesarean section; FGR, fetal growth restriction, GA, gestational age; GDM, gestational diabetes; GH, gestational hypertension; HG, hyperemesis gravidarum; HUS, hemolytic uraemic syndrome; MFPR, multifetal pregnancy reduction; PPH, postpartum hemorrhage; PTB, preterm birth; VD, vaginal delivery.

### Main themes

**Figure 1.**
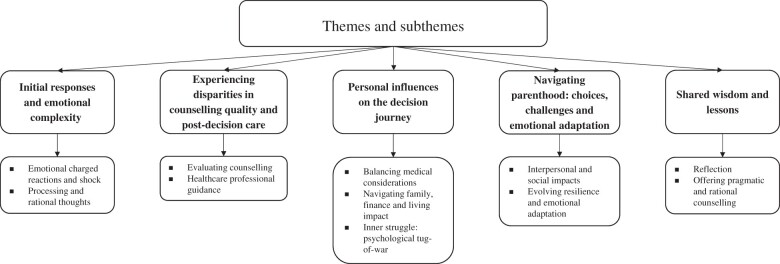
Themes and subthemes.

Five main themes were identified and several subthemes derived from the main themes (see Fig. 1).

#### Theme 1: initial responses and emotional complexity

The ultrasound revealing a triplet pregnancy consistently elicited emotionally charged reactions and shock among the fathers.

‘*…, we were overwhelmed*.’ (MFPR group)

‘*Then my whole life flashed by…*’ (MFPR group)

A complex interplay of emotions emerged during this revelation. Some fathers started laughing and crying at the same time. A few were very happy, and others were speechless.

‘*Number one, number two, and then we also saw number three. Yes, then we fell silent*.’ (ongoing triplet group)

‘*We were also feeling euphoric: a triplet!*’ (ongoing triplet group)

Contrarily, one father stated:

‘*My life is over.*’ (MFPR group)

Many fathers reported rational thoughts. These revolved around pragmatic considerations, such as the need for a bigger car, moving to a more spacious house, or whether they had the financial means for raising three children simultaneously.

‘*Being a man, you go in the practical modus immediately.*’ (ongoing triplet group)

The mixture of emotional shock and practical consideration emphasized the initial responses of the fathers, revealing a complex interplay between their emotional and rational faculties.

#### Theme 2: experiencing disparities in counselling quality and post-decision care

All fathers received counselling before deciding whether to perform MFPR, primarily within a tertiary care hospital setting, although some continuing triplets received this information elsewhere.

Notably, the majority of the fathers had never heard of MFPR before, as highlighted by one father that stated:

‘*… it is desirable to inform parents about the consequences of a multifetal pregnancy and the option of reduction earlier than the moment the triplet pregnancy reveals, for example during a fertility treatment.*’ (ongoing triplet group)

The quality of the counselling received varied among the participants. Some fathers expressed contentment with the provided information.

‘*The information was excellent, very clear.*’ (MFPR group)

‘*She explained it all very clear and calm.*’ (MFPR group)

‘*I cannot think of any information that should have been added to make a more informed decision.*’ (MFPR group)

In contrast, others were less content.

‘*… estimating percentages. That is not very specific and concrete.*’ (MFPR group)

‘*There was nothing, not even a brochure.*’ (ongoing triplet group)

‘*I needed to search on google, to obtain more information.*’ (MFPR group)

‘*There was the opportunity to ask for the risks, but there was only little research about the topic.*’ (ongoing triplet group)

‘*We searched for scientific articles by ourselves.*’ (ongoing triplet group)

Interestingly, a father stated that the provided information was mainly focused on specific aspects, such as the chance of survival of the children. He reported:

‘*…, but there was no information about what you can expect when you are having three children all at once. How do you manage something like that? How are you going to combine that with your work, especially when they will be born prematurely? What will happen after birth? … I needed more information on that. What kind of setbacks can you expect? Then you can prepare your network and your work better.*’ (ongoing triplet group)

In terms of risk awareness, fathers in the MFPR group were aware of the small risk of losing the pregnancy after the procedure. However, those in the ongoing triplet group did not seem to have appropriate knowledge or were unconscious about the risks associated with maintaining a triplet pregnancy, such as the consequences of early preterm birth (e.g. having a child with disabilities or perinatal death).

After the decision, some couples were referred back to secondary care, since twin pregnancies or triplet pregnancies with a gestational age of >32 weeks can be under care and delivered in a secondary care hospital. Several fathers reported on insufficient care during pregnancy.

‘*We may have seen the same doctor only once.*’ (ongoing triplet group)

‘*You need to have a more fixed guidance trajectory. This can be with a doctor, but can also be with a nurse, or resident.*’ (ongoing triplet group)

After childbirth, none of the fathers were offered a follow-up appointment by the hospital where the counselling of MFPR took place. Most interviews revealed a significant need for aftercare. This underscores a potential gap in the continuity of support provided by healthcare institutions.

#### Theme 3: personal influences on the decision journey

This theme explores the multifaceted considerations guiding fathers as they need to decide together with the mother between MFPR and maintaining the triplet pregnancy (Table 4). Three distinct subthemes emerge: (i) balancing medical considerations, emphasizing factors like obstetrical history, the mother's physical state, and the risks associated with MFPR and maintaining the triplet pregnancy; (ii) navigating family, finance, and living impact, highlighting the dominance of social factors, including discussions about family dynamics, financial stability, living conditions, and the opinions of family members; and (iii) inner struggle: psychological tug-of-war, revealing a complex interplay between emotional and rational factors.

**Table 4.** Personal influences on the decision journey.

Topics per subtheme	Quotes
**Balancing medical considerations**	
Obstetrical history	‘*We had been trying to conceive already for so long,…*’ (ongoing triplet group) ‘*… if it was our first pregnancy, then we would have kept the triplet…*’ (MFPR group)
First trimester findings such as type of pregnancy (trichorionic/dichorionic), result of ultrasound, result of NIPT	‘*…, they all had their own sac, their own amniotic fluid, so everything went as it should.*’ (ongoing triplet group) ‘*All three were looking healthy.*’ (ongoing triplet group) ‘*… it was a major requirement that the NIPT was good.*’ (ongoing triplet group)
Physical state of the mother	‘*Maybe her body is not strong enough.*’ (MFPR group) ‘*… I know she is a strong person, also physically, why wouldn’t we do it?*’ (ongoing triplet group)
Risk of MFPR	‘*… this is a risk, it can be true that you lose the whole pregnancy.*’ (ongoing triplet group)
Risk of maintaining triplet pregnancy	‘*I don’t want to raise a disabled child,…*’ (MFPR group) ‘*What are the risks for the mother? For me, that’s more important than the risks for the babies.*’ (ongoing triplet group)
**Navigating family, finance and living impact**	
Financial status	‘*…, can we do it from the economic point of view?*’ (MFPR group) ‘*Economic interest seems to be a bottom priority,…*’ (ongoing triplet group) ‘*When in Africa they are able to raise children without appropriate resources, then we are also able to do it here, with all the luxury that we have here.*’ (ongoing triplet group)
Living situation	‘*… then we should give up everything and move to… [place in the Netherlands].*’ (MFPR group)
Consequences for other child(ren)	‘*… [other child] would receive much less attention.*’ (MFPR group)
Opinion of the mother	‘*… my wife had a lack of trust that she could deliver three healthy babies, that was her biggest fear.*’ (MFPR group) ‘*If it was up to me, then we had removed one child. For my partner, this was absolutely not an option. She would never take away children.*’ (ongoing triplet group)
Involving other people	‘*We talked to nobody other than each other.*’ (MFPR group) ‘*…, we talked a lot about it with our family.*’ (ongoing triplet group) ‘*… everybody panicked.*’ (ongoing triplet group)
**Inner struggle: psychological tug-of-war**	
Emotional	‘*How will you feel if you have two children, and you know that there was a third …*’ (ongoing triplet group) ‘*… I had the fear that in the end it wouldn’t go well.*’ (MFPR group) ‘*How are we going to do it with three children, as one was really hell already.*’ (ongoing triplet group) ‘*In the end, we followed our heart instead of our head.*’ (ongoing triplet group)
Rational	‘*… in the end, it is her body.*’ (MFPR group) ‘*I’m just very sober, for me it is no child yet.*’ (ongoing triplet group)

MFPR; multifetal pregnancy reduction; NIPT, non-invasive prenatal test.

#### Theme 4: navigating parenthood: choices, challenges, and emotional adaptation

In terms of interpersonal and social impacts, practical, relational, and financial challenges were discussed. Fathers of both groups declared that they received help in their household such as support from family members, friends, and/or a nanny.

‘*Without your network, you do not stand a chance.*’ (ongoing triplet group)

‘*… you are always short of hands.*’ (ongoing triplet group)

Several fathers of the ongoing triplet group experienced a significant influence on the relationship with their partner, with some referring to their household as a *‘factory’*.

‘*… it is all about raising your children, that’s a consequence of having triplets.*’ (ongoing triplet group)

‘*It is like a military operation when you are raising triplets.*’ (ongoing triplet group)

‘*Time for each other, that is something that we really need to schedule*’. (ongoing triplet group)

Others experienced raising triplets as strengthening for their relationship.

‘*It has brought us closer together.*’ (ongoing triplet group)

In all interviews, the concept of developing resilience and adapting emotionally took a prominent role. Several fathers became emotional during the interviews since they experienced the decision as difficult and very impactful.

‘*This was by far the most difficult decision in my whole life.*’ (ongoing triplet group)

Fathers of the MFPR group expressed feelings of grief to different levels. One father declared he was ‘*still struggling*’ with ‘*letting go of a child*’, and proclaimed:

‘*…, truly without exaggerating, I think about it every week.*’ (MFPR group)

One father stated that the procedure of MFPR felt like ‘*murder*’, and like ‘*taking a life*’. Another father still thought about the moment of the reduction. Being able to watch the ultrasound during the reduction; he could still hear ‘*the heartbeat fading away*’.

‘*… I will never forget it.*’ (MFPR group)

Another father stated:

‘*… this [MFPR] could be so traumatic that you suffer from it your whole life.*’ (MFPR group)

While grieving or experiencing regret over the loss of one or more fetuses, fathers after MFPR also felt a sense of relief due to reducing the risks associated with triplet pregnancies. They expressed that they would make the same decision if faced with it again, although some still struggled with the emotions related to the MFPR decision. Some indicated that they did not talk about it very often; one father did not talk about the reduction to his social or family network at all. Not all fathers had found a suitable way to process the emotions related to the procedure, but some were trying:

‘*… I asked a friend who is painter if he maybe could make a painting with two adults and two children watching a star,…*’ (MFPR group)

In the ongoing triplet group, some fathers experienced stress and fear during pregnancy or birth due to medical complications. One father stated that he thought he was ‘*basically going to lose the children*’. Another experienced a shift in his role and responsibilities as a result of extended hospitalization of both the mother and children, leading him to reflect on his position within the family and towards his partner. Several fathers of the ongoing triplet group mentioned feelings of mental and emotional exhaustion during the early years of raising triplets.

‘*You are in a continuous state of hyperactivity, … that is not healthy for body and mind.*’ (ongoing triplet group)

‘*There is not much space for emotions.*’ (ongoing triplet group)

‘*…, I had a small burn-out.*’ (ongoing triplet group)

However, at the time of the interview, most fathers were *‘very happy’* with their triplets. They noted that raising them is a unique challenge and a positive and fulfilling experience. One father of the ongoing triplet group would have chosen for MFPR when he could decide again. In terms of relational agreements, all fathers who participated reported that they agreed with their partner about the decision.

#### Theme 5: shared wisdom and lessons

Within this theme, fathers offered valuable reflections and guidance to those facing similar situations. Reflective insights included rational advice wherein participants were encouraged to search for information apart from what is provided during formal counselling.

A father emphasized his preference to ‘*always primarily decide rationally*’, underlining the importance of an informed approach. Additionally, fathers suggested visiting families who are raising triplets to gain first-hand perspectives.

Emotional advice centred on guiding others to navigate the process of deliberating and processing the decision.

‘*Make sure that you go through the decision-making process with full commitment and consciousness, … It is the most important decision of your life.*’ (ongoing triplet group)

The emotional dimension also prompted advice about open communication with relatives. One father declared:

‘*It is a life-changing moment, everything is upside down, … Do you have someone to talk to? You cannot do it alone.*’ (ongoing triplet group)

These insights and counsel together form a portrayal of the father’s journeys, offering reflective contemplation with practical recommendations for those undertaking similar parts.

### Telephone appointment after interview

All fathers reported a positive experience discussing the subject, finding it helpful in handling the process. None of the fathers expressed a need for additional aftercare arranged by the study team.

## Discussion

In this qualitative study, we found that for fathers of triplet pregnancies, the decision whether to reduce or maintain a triplet pregnancy is complex, in which medical, psychological, but mainly social factors play an important role. In terms of consequences, particularly the psychological impact and consequences of the decision are extensive. Fathers after MFPR can still struggle with difficult emotions towards the decision and express feelings of doubt or regret years after the procedure. Fathers raising triplets experience this as a physically and emotionally demanding process as a result of increased stress during the first years after birth, with consequences for their mental health. In the end, the majority of the fathers feel that they have made the right decision, except for one father who regretted the decision to maintain the triplet pregnancy. In terms of aftercare, there was a discrepancy between need and offer. However, all fathers seemed reasonably adjusted, and there was no need of support at the time of the interview.

This study contains an overview of the decision-making process of MFPR, and the consequences of the decision in fathers from medical, psychological, and social perspectives based on qualitative analysis, which is a comprehensive approach that enables in-depth exploration of our fathers’ perspectives.

The findings of this study should be interpreted within certain limitations. First, while this qualitative study successfully reached data saturation, it is important to acknowledge the relatively small sample size. The depth and complexity of the participants' responses contributed to reaching saturation within this limited sample. Consequently, this study can serve as inspiration for further research and exploration in the field. Second, while our current study focused on the specific MFPR process in the Amsterdam region, we recognize the importance of exploring how this process may differ across other regions in The Netherlands and internationally. We emphasize the need for caution in generalizing our findings beyond the Amsterdam region. We believe that additional research, particularly comparative studies involving different regions and countries, would contribute valuable insights into the variations in care processes and decision-making experiences related to MFPR. Third, the study findings might be limited due to selection bias, since fathers with less problems and relatively positive stories may have been more willing to participate. This is reflected by the successful pregnancy outcomes of the fathers who participated in this study; the pregnancies continued to 30 weeks of gestational age and beyond, and all neonates were liveborn and survived (Table 3). Thus, the findings might not fully capture the range of experiences and perspectives within the target population. Contrary, fathers with mental health symptoms, or milder forms within the normal spectrum of human emotions, may be more inclined to participate to seek a supportive environment to share their experiences and feelings. Fourth, in this study, the mothers were the intermediaries in the recruitment process of the fathers, potentially leading to a selective engagement of fathers who display greater levels of cooperation. Consequently, fathers who are less involved or separated (such as through divorce) from the mothers might be underrepresented in this sample which is limiting the generalizability of our findings. Fifth, the interviews were held from 1 to 6 years after the decision whether to perform MFPR, which may have influenced the fathers’ emotional processing. However, there is no fixed timeline or specific number of years after a life event that can overcome this issue, since emotional processing is a subjective and individual process that can persist for years or even a lifetime. Additionally, several fathers displayed emotional responses regardless of the time elapsed since the decision. A last limitation inherent to this study is the potential for interviewer bias, deriving from the authors' underlying philosophical assumptions that may influence data interpretation. However, as detailed in the methods section, the interviewers actively participated in reflective discussions to mitigate any such influence and uphold the integrity of the study's findings.

No previous studies reported on the impact of MFPR or maintaining triplets on fathers exclusively, and therefore the findings of this study contribute to the existing knowledge in this area. It is crucial to raise awareness about the significant emotional impact of the decision which is a stressful life event that can have long-lasting repercussions. Psychological consequences may vary depending on individual differences in beliefs, coping styles, social support, and previous psychological history. Addressing the psychological consequences requires profound cognitive processing and the creation of meaning. It is important to consider the individual experiences and needs of fathers and provide appropriate support as part of a multidisciplinary approach of care for parents with multifetal pregnancies. Lack of support seems to maximize the potential of such event to have traumatic repercussions and this should be avoided. Seeking professional support can be beneficial to fathers who experience emotional distress or other psychological challenges related to either MFPR or maintaining the multifetal pregnancy. Therefore, it is of great importance that healthcare providers offer appropriate aftercare. Then, they can provide a more tailored guidance and support which can help parents experiencing emotional difficulties.

## Conclusion

The emotional impact of either MFPR or caring triplets is extensive and requires careful consideration. It is crucial to increase awareness among caregivers about the emotional challenges faced by fathers. A guided trajectory might be needed that includes comprehensive counselling, informed decision-making support, and appropriate aftercare, not only for the mother but also for the father. This might optimize the decision-making process and outcomes of fathers after MFPR or caring triplets, including tailored care to fathers who may be experiencing emotional distress or other psychological challenges. Care might help with processing the event and the decision made, to promote the construction of positive meaning around a decision no one asks to do but cannot be avoided and has a traumatic potential. Thus, healthcare providers can play a vital role in promoting the mental health and well-being of fathers and their families.

## Supplementary Material

dead275_Supplementary_Table_S1

## Data Availability

The data underlying this article will be shared on reasonable request to the corresponding author.
