# Supplementary material for: Considering multifetal pregnancy reduction in triplet pregnancies: do we forget the emotional impact on fathers? A qualitative study from The Netherlands
Source: Hum Reprod. 2024 Jan 10;39(3):569–77. doi: 10.1093/humrep/dead275 (PMC10905497; doi:10.1093/humrep/dead275)
Supplement: dead275_Supplementary_Table_S1 [file dead275_supplementary_table_s1.pdf]

OXFORD
human reproduction
Human Reproduction, 2024, 1–1
https://doi.org/10.1093/humrep/dead275
Supplementary Data

Supplementary Table S1. Conducted interviews, interviewers, and analyzers.

Participant	Interviewers	Analyzers
**P1.**	P.M.v.B. and M.G.v.P.	P.M.v.B., W.F.J.G., and M.G.v.P.
**P2.**	P.M.v.B.	P.M.v.B., W.F.J.G., and M.G.v.P.
**P3.**	P.M.v.B.	P.M.v.B., W.F.J.G., and M.G.v.P.
**P4.**	P.M.v.B.	P.M.v.B., W.F.J.G., and M.G.v.P.
**P5.**	P.M.v.B. and M.G.v.P.	P.M.v.B., W.F.J.G., and M.G.v.P.
**P6.**	P.M.v.B. and W.F.J.G.	P.M.v.B., W.F.J.G., and M.G.v.P.
**P7.**	M.G.v.P. and W.F.J.G.	P.M.v.B., W.F.J.G., and M.G.v.P.
**P8.**	P.M.v.B. and W.F.J.G.	P.M.v.B. and M.G.v.P.
**P9.**	P.M.v.B., W.F.J.G., and M.G.v.P.	P.M.v.B. and M.G.v.P.
**P10.**	P.M.v.B. and W.F.J.G.	P.M.v.B. and M.G.v.P.
**P11.**	P.M.v.B. and W.F.J.G.	P.M.v.B. and M.G.v.P.
**P12.**	P.M.v.B. and W.F.J.G.	P.M.v.B. and M.G.v.P.
